# Feasibility of challenging treadmill speed-dependent gait and perturbation-induced balance training in chronic stroke patients with low ambulation ability: a randomized controlled trial

**DOI:** 10.3389/fneur.2023.1167261

**Published:** 2023-07-17

**Authors:** Jia Hu, Lingjing Jin, Yubing Wang, Xia Shen

**Affiliations:** ^1^Medical Education Department, Shanghai YangZhi Rehabilitation Hospital (Shanghai Sunshine Rehabilitation Center), School of Medicine, Tongji University, Shanghai, China; ^2^Rehabilitation Medicine Research Center, Shanghai YangZhi Rehabilitation Hospital (Shanghai Sunshine Rehabilitation Center), School of Medicine, Tongji University, Shanghai, China; ^3^Department of Rehabilitation Sciences, Tongji University School of Medicine, Shanghai, China

**Keywords:** stroke, gait, balance, perturbation, treadmill, velocity, intensity

## Abstract

**Background:**

Treadmill training shows advantages in the specificity, amount, and intensity of gait and balance practice for the rehabilitation of stroke patients.

**Objective:**

To investigate the feasibility and effectiveness of challenging treadmill speed-dependent gait and perturbation-induced balance training in chronic stroke patients with low ambulation ability.

**Methods:**

For this randomized controlled trial (Chinese Clinical Trials.gov registration number ChiCTR-IOR-16009536) with blinded testers, we recruited 33 ambulatory stroke participants with restricted community ambulation capacity and randomly assigned them into two groups: the experimental group with 2 week treadmill speed-dependent gait training combined with 2 week treadmill perturbation-induced balance training (EXP) or the control group with traditional gait and balance training (CON). Various variables were recorded during EXP training, including the rating of perceived exertion, heart rate, causes of pauses, treadmill speed, and perturbation intensity. Outcome measures were examined before training and at 2 and 4 weeks after training. They included gait velocity during five-meter walk test at comfortable and fast speed and reactive balance ability in the compensatory stepping test as primary outcome measures, as well as dynamic balance ability (timed up-and-go test and 5 times sit-to-stand test) and balance confidence as secondary outcome measures.

**Results:**

All participants completed the study. The treadmill speed and perturbation intensity significantly increased across training sessions in the EXP group, and no adverse effects occurred. The normal and fast gait velocities showed significant time and group interaction effects. They significantly increased after 2 and 4 weeks of training in the EXP group (*p* < 0.05) but not in the CON group (*p* > 0.05). Likewise, dynamic balance ability measured using the timed up-and-go test at a fast speed significantly improved after 2 and 4 weeks of training in the EXP group (*p* < 0.05) but not in the CON group (*p* > 0.05), although without a significant time and group interaction effect. Surprisingly, the reactive balance ability did not show improvement after treatment in the EXP group (*p* > 0.05).

**Conclusion:**

Challenging treadmill speed-dependent gait and treadmill perturbation-induced balance training is feasible and effective to improve ambulation function in chronic stroke patients with low ambulation ability.

## 1. Introduction

Gait impairments are major disabling disorders in hemiplegic patients after stroke, which greatly compromises their ambulation independence, increases the risk of falls, and reduces quality of life (1–4). Restoration of walking ability is the hallmark of rehabilitation for these patients (2, 5). However, the recovery of walking ability reaches a plateau at the chronic stage, usually around 6 months after stroke (4, 6–8), when most patients achieve independent ambulation but suffer considerable gait deficits, including slow gait speed and poor postural stability, restricting their community ambulation (8–10).

With little spontaneous recovery, therapeutic-induced plasticity is the main mechanism underlying the resumption of activity and function at the chronic stage of stroke recovery (11). The specificity, amount, and intensity of walking practice are crucial variables of the treatments that can facilitate plasticity of neuromuscular and cardiopulmonary systems and ensure improvement of walking ability (12).

In a traditional session of physical therapy, gait training is usually performed overground and combines a series of balance exercises, emphasizing a normal pattern and stability during walking practice and commonly using some facilitation techniques such as the Bobath concept (2, 5). The traditional training method limits the amount of walking practice to fewer than 200 repetitions of stepping for ambulatory patients (13). That does not meet the requirements for motor learning and neural plasticity to occur (14, 15) and restrains the intensity in terms of walking speed during training. Thus, an insufficient amount and intensity of walking practice could be a critical cause of suboptimal effectiveness in traditional intervention for stroke patients at the chronic stage.

Treadmill-based exercise is a popular training method for ambulation function in patients after stroke (2). This modality delivers three-fold more step practice than traditional training within the same time for stroke patients (16, 17). It can be performed with or without body weight support or the use of handrails and with adjustable speed to ensure both safety and intensity of gait training in stroke patients with different degrees of gait deficits (18). Body weight support and the use of handrails provide physical assistance to exerciser, can decrease balance control demand during walking, thereby improving gait performance in kinematics and promoting gait economy (19, 20). However, physical assistance reduces the balance training component and might lower the intensity of walking exercises to a point that is insufficient for the production of biomarkers involved in neuromuscular plasticity, such as lactate and brain-derived neurotrophic factor (21, 22).

Ample evidence has shown that compared with traditional overground walking, treadmill gait training without body weight support, but not that with, has superior benefits in improving walking performance in patients with independent ambulation (18). Furthermore, higher speed during treadmill training such as speed-dependent training mode, no matter with or without body weight support, improves the walking performance of stroke patients more than lower-speed training mode, such as using a comfortable speed (23–25). The evidence demonstrates the importance of challenging training protocols in optimizing training efficacy. Moreover, we noted that handrails were commonly used or permitted for stroke patients to warrant safety during treadmill training without weight support, independent of speed intensity (18). For chronic stroke patients who already achieved independent ambulation but remain restricted in community ambulation, we supposed that handrails might not be necessary. Therefore, we proposed a challenging treadmill speed-dependent gait training protocol with neither weight support nor handrail use.

Poor postural stability is a prominent feature involved in hemiplegic gait deficit and increases the risk of falling in stroke patients (26). An efficient reactive balance response is crucial to prevent stroke patients from falling when experiencing loss of balance. However, impaired reactive balance ability is common in stroke patients and considerably contributes to their increased fall rates during inpatient rehabilitation and after returning to the community (1, 27). Thus, balance training targeting anticipatory and reactive postural control is commonly integrated into gait training in traditional physical therapy sessions for stroke patients. In traditional balance training protocols, anticipatory postural control is usually practiced through a series of balance-specific activities such as standing, stepping, and walking; reactive postural control is typically trained by manual perturbation during balance tasks (5, 28–31).

The abovementioned treadmill gait training consistently generates a backward-directed force to the patient’s sole during walking that disturbs the balance to simultaneously train the anticipatory postural control. We expected that the speed-dependent training mode without physical assistance is superior in both dose and intensity than traditional balance training for training the anticipatory gait-related postural control. For reactive postural control, a treadmill-based training mode has been recently reported (28, 32), which is innovative compared with traditional manual perturbation training (29–31). It simulates the unpredictable perturbation of falls in real life such as slips, trips, or pushes during normal walking by belt acceleration, deceleration, and treadmill platform lateral movement, respectively (28, 32). The innovative treadmill perturbation-induced training has shown effective to improve reactive balance ability (28, 32), has superior benefits in improving gait performance compared with treadmill gait training (32), and exhibited greater effects on increasing balance confidence compared with traditional gait training in stroke patients with relatively high ambulation capacity who already have community ambulation capacity. We expected the training mode is feasible and effective in stroke patients with low ambulation ability as well, who are still restricted to community ambulation although they are already capable of independent ambulation.

To summarize, treadmill-based training shows advantages in the specificity, amount, and intensity of walking and balance practice for the rehabilitation of stroke patients. Taking safety preconditions into account, a sufficiently challenging treadmill gait and balance training protocol is the key to optimizing the treatment efficacy for ambulation function. For chronic stroke patients with low ambulation ability, we proposed a challenging gait and balance training protocol combining treadmill speed-dependent gait training with perturbation-induced balance training during treadmill walking.

The objectives of the present study were to investigate whether the challenging treadmill gait and balance training is feasible for chronic stroke patients with low ambulation ability and to explore the effects on improving ambulation function compared to traditional gait and balance training in those patients. We hypothesized that it is feasible and effective in chronic stroke patients with low ambulation ability.

## 2. Methods

### 2.1. Participants

Patients admitted to the Shanghai Yangzhi Rehabilitation Hospital (Shanghai Sunshine Rehabilitation Center) between May 2018 and December 2019 were enrolled in this study (Table 1). Inclusion criteria included patients with a first-episode stroke, a disease course longer than 6 months, perceived balance impairment with impact on daily life (Affirmation to the question: “Do you feel that you experience balance impairment in your daily living?”), the ability to walk independently with or without an assistive device for at least 10 meters, and gait velocity lower than 0.66 m/s. The gait velocity of 0.66 m/s is a recognized cutoff point for community ambulation capacity (10). Exclusion criteria included patients with skeletal injuries or diseases that affected gait and balance ability, including recent fracture of the trunk or lower limbs, severe osteoarthritis or osteoporosis, vision impairment and neglect, unable to understand verbal commands (who were screened using the 3-step command task of the Mini-Mental State Examination), unstable blood pressure control, and severe cardiopulmonary disease, which was checked by medical record at the time of enrollment.

**Table 1:** The table of perturbation intensity and corresponding translation movement parameters.

Perturbation intensity	Forward-backward translation			Lateral translation		
	Amplitude	velocity	Acceleration	Amplitude	velocity	Acceleration
	(cm)	(cm/s)	(cm/s^2^)	(cm)	(cm/s)	(cm/s^2^)
1	1	4	13	1	4	13
2	2	7	26	1.6	6	20
3	3	11	38	2.2	8	28
4	4	14	51	2.8	10	35
5	5	18	64	3.3	12	43
6	6	21	77	3.9	14	50
7	7	25	89	4.5	16	58
8	8	29	102	5.1	18	65
9	9	32	115	5.7	20	73
10	10	36	128	6.3	22	80
11	11	39	140	6.9	25	88
12	12	43	153	7.4	27	95
13	13	46	166	8	29	102
14	14	50	179	8.6	31	110
15	15	54	191	9.2	33	117
16	16	57	204	9.8	35	125
17	17	61	217	10.4	37	132
18	18	64	230	11	39	140
19	19	68	242	11.5	41	147
20	20	71	255	12.1	43	155
21	21	75	268	12.7	45	162
22	22	79	281	13.3	48	170
23	23	82	293	13.9	50	177
24	24	86	306	14.5	52	185
25	25	89	319	15.1	54	192
26	26	93	332	15.7	56	200
27	27	96	344	16.2	58	207
28	28	100	357	16.8	60	215
29	29	104	370	17.4	62	220
30	30	107	383	18	64	230

To estimate the sample size, we referred to a previous study comparing innovative treadmill gait training with overground gait training in chronic stroke patients with independent ambulation but still restricted community ambulation (33). An effect size of 1.0 for the difference of change in comfortable gait velocity between two training modes was calculated based on this previous study. Setting a significance level of 0.05 and a power (1−β) of 0.8, we estimated that 17 patients in each group were required for significant analyses using the *t*-test. Considering a 15% dropout rate, the initial sample size was set at 20 in each group.

The study conformed to the principles of the Declaration of Helsinki and was approved by the Medical Ethics Committee of the Guangdong Work Injury Rehabilitation Center (AF/SC-07/2016.01, Chinese Clinical Trials.gov registration number ChiCTR-IOR-16009536). The ethical approval was endorsed by the ethics committee of Shanghai Yangzhi Rehabilitation Hospital (Shanghai Sunshine Rehabilitation Center). All eligible participants were enrolled after providing written informed consent.

### 2.2. Study design

All participants were randomly assigned into one of two groups by drawing lots from sealed envelopes: The experimental group with challenging treadmill gait and balance training (EXP) and the control group with traditional gait and balance training (CON). All participants were assessed three times consecutively after enrollment (before training, and at 2 weeks and 4 weeks after training) by two examiners who were unaware of the grouping. All assessments were carried out by the same examiners.

Participants in both the EXP and CON groups received physical therapy for 4 weeks, with five sessions per week and 1.5 h per session, where the therapist conducted 30 min of one-on-one gait and balance training. Patients in the CON group received 4 weeks of traditional gait and balance training, 5 sessions per week. Patients in the EXP group received treadmill speed-dependent gait training for the first 2 weeks, followed by perturbation-induced balance training during treadmill walking at a comfortable speed for the second 2 weeks, with three sessions per week. This treadmill training frequency showed an improved time efficacy ratio than others (18). To match the training duration between the two groups, patients in the EXP group additionally received two sessions of traditional gait and balance training per week for 4 weeks.

### 2.3. Challenging treadmill gait and balance training

The EXP training was conducted using a movable treadmill system (Balance Tutor, Medi Touch, Ltd., Israel). Patients wore a harness attached to an overhead sling system for fall prevention but without any body weight support throughout the training sessions. Handrail use was not allowed during the training sessions, but assistive devices for daily use were permitted if needed. The rating of perceived exertion (RPE; ranging from 0 to 10) and the heart rate were monitored before and during the training sessions.

Unlike traditional approaches that mix gait and balance training in one session, we separated the treadmill speed-dependent gait training from the treadmill perturbation-induced balance training in the first and second 2 week phases, respectively, based on two considerations. First, we aimed to clarify the feasibility of each training mode for chronic stroke patients with low ambulation ability. Separating the two training modes allowed this to be accomplished. Second, owing to overall safety considerations, our objective, in the first phase of gait training, was to initially improve ambulation function to some extent. This was expected to develop the abilities of patients in the perturbation-induced balance training.

For the treadmill speed-dependent gait training during the first 2 weeks, the comfortable speed and the initial maximum speed on the treadmill were determined before the first training session. Each treadmill speed-dependent gait training session consisted of one 5 min warm-up phase, four phases of 5 min treadmill speed-dependent gait training, and one 5 min cool-down phase in the end. During the warm-up and cool-down phases, the participant walked at the comfortable speed. During the speed-dependent training phases, the initial maximum speed was used as the first 5 min speed-dependent training. If the participant could continuously walk without loss of balance for 5 min, the RPE after 5 min phase did not reach 6/10, and the heart rate did not exceed the safe heart rate of 80% heart rate reserve [(220 − resting heart rate) × 80% + resting heart rate], then the speed was increased by 0.2 km/h every 5 min. In contrast, if the participant could not walk at the initial maximum speed safely (loss of balance, interruption, RPE ≧6/10, or heart rate exceeding the safe heart rate, or any discomfort too severe to continue), rest was provided until they felt well, and then the speed was reduced by 0.2 km/h for the next 5 min training phase. The maximum speed in each training session was recorded and used as the initial maximum speed for the next gait training session. Participants were encouraged to walk at a fast speed in daily life.

For the perturbation-induced balance training during the second 2 weeks, the comfortable walking speed and the initial intensity threshold of perturbations in each of four directions to trigger a stepping response while walking at the comfortable speed were determined before the first training session. The intensity of perturbation generated by the treadmill platform ranges from 1 to 30 levels based on the amplitude, velocity, and acceleration of the induced movement of the platform. Surface translations were increased systematically in perturbation trials (characteristics of perturbation intensities are described in Table 1). Each treadmill perturbation-induced balance training session consisted of one 5 min warm-up phase, four phases of 5 min perturbation-induced balance training while walking at the comfortable speed, and one 5 min cool-down phase in the end. During the warm-up and cool-down phases, the participant walked at the comfortable speed without perturbation. During the perturbation-induced training phases, the treadmill belt or platform introduced a perturbation in a random direction about every 10 s at the previously tested threshold intensity, no matter which phases of the walking cycle (28, 32). Perturbations in all four directions were elicited: forward translational perturbation like slipping triggered by the acceleration of the treadmill belt, backward translational perturbation like tripping triggered by deceleration of the treadmill belt, and left or right translational perturbation like a push at the foot level by lateral acceleration of the movable treadmill platform. The participant was encouraged to make a stepping reaction to enlarge the base of support and follow the moving belt, using the leg with no or less weight bearing which could be either the paretic or the non-paretic leg.

If the participant performed proper stepping reactions and continued to walk safely without interruption or loss of balance, with an RPE ≦ 5/10, with a heart rate not exceeding the safe heart rate, and without discomfort, then the perturbation intensity was increased by 2 levels every 5 min. If the participant made an improper stepping reaction but continued to walk safely, the current perturbation intensity was maintained, but visual or auditory cues were provided to remind the participant of proper stepping responses until they performed them properly. If the translational perturbation or walking resulted in the loss of balance, an RPE ≧6/10, a heart rate exceeding the safe heart rate, or any discomfort too severe to continue, rest was provided until the participant felt well, and then the perturbation intensity was reduced by 2 levels in the next training phase; visual or auditory cues were given as well to remind the participant to make correct responses. The maximum intensity of perturbation in each direction in each training session was recorded and used as the initial perturbation intensity for the next perturbation-induced training session. The participants were encouraged to make reactive stepping responses when encountering perturbation in daily life.

### 2.4. Traditional gait and balance training

The training targeted a series of functional tasks, including standing under different conditions (with reduced sensory input, with a reduced base of support, while reaching or catching some target, or responding to unexpected manual perturbation), transferring from sitting to standing, multidirectional stepping, overground walking, and going up and down stairs. Patients were allowed to use their assistive devices, if needed. The stability and normal pattern of weight bearing and movements during tasks was emphasized during training. The participants were encouraged to use the postural control and movement strategies learned from the training in daily activities. Each session lasted for 30 min.

### 2.5. Assessment protocol

We assessed a series of functional outcomes before training (Pre), 2 weeks after training (Mid), and 4 weeks after training (Post). All participants completed all tests independently without any assistive device except for one participant in the CON group who wore an ankle-foot orthosis during tests at each assessment.

As an efficient intervention should have effects that transfer across dimensions of health in routine environments (34), we adopted two primary outcomes and three secondary outcomes across body function, activity, and participation dimensions, all of which were tested on the ground but not on the treadmill.

### 2.6. Primary outcomes

The two primary outcomes measured were gait velocity and reactive balance ability.

The five-meter walk test was used to examine the comfortable and fast gait velocities. This test has excellent reliability and validity in patients with stroke (35, 36). For the test, a nine-meter-long walkway was set consisting of five meters in the middle and two meters at each end. The participant was asked to walk from one end of the nine-meter-long walkway to the other end. The time spent during the middle five-meter walk was recorded to calculate the velocity. The test was repeated twice at the participants’ comfortable speed and at their fastest speed after one practice round, and the mean values of the two tests at each condition were used for analysis. The minimal clinically important difference is 0.05 m/s for stroke patients (37).

The forward and backward compensatory stepping test (CST) items of the Balance Evaluation Systems Test, a reliable test in patients with hemiplegia (38, 39), were adopted to examine the reactive balance ability. During the forward CST, the participant stood with their feet shoulder width apart and arms at their sides, then leaned forward against the tester’s hands beyond the forward limits (the hips are in front of the toes), then the participant was asked to do whatever is necessary to avoid a fall when the tester released their hands, including making a stepping response (38). The backward CST had a similar procedure except that the participant leaned backward beyond the backward limits (the hips are behind the heels) by making a backward stepping response. The items were scored on a 4-point scale from 0 to 3 indicating severe impairment, moderate impairment, mild impairment, and normal, respectively (38). The tests were conducted at least once after the participant understood the procedure from observing the demonstration. For participants who maintained their balance by adjusting their posture using a non-stepping strategy, more test trials were provided to guide them to lean more or to increase the pushing force against them, until the participant made a stepping response or fell without stepping (38).

### 2.7. Secondary outcomes

The timed up-and-go test (TUG) at the comfortable and fastest speeds, and the 5 times sit-to-stand test (5-STS) were used to examine the dynamic balance ability. The activities-specific balance confidence (ABC) scale was used to examine balance confidence of the participants. These scales have demonstrated strong test–retest reliability in patients with stroke (40–42).

Besides the functional outcomes, a series of variables monitored during the challenging treadmill gait and balance training were recorded as well. These included the maximum RPE and heart rate during each training session, the maximum speed during each speed-dependent gait training session, and the maximum intensity of perturbation in each direction during each perturbation-induced balance training session. Furthermore, demographic and disease features were obtained upon enrollment from the participants and confirmed based on their medical record, which included sex, age, height, weight, type of stroke, time since stroke, hemiplegia side, and the Brunnstrom stages of the lower extremity.

### 2.8. Statistical analysis

The SPSS 20.0 software was used for statistical analysis. The Shapiro–Wilk test was used to confirm the normal distribution of data. The independent *t*-test or Pearson χ^2^ was used to analyze between-group differences in demographic and disease characteristics, and functional outcomes at baseline, for variables with normal distribution and those without normal distribution, respectively. The two-way (time and group) repeated measures analysis of variance (ANOVA) test for variables of functional outcomes with normal distribution was used to analyze between-group differences in training effects, with any demographic and disease characteristics as covariates if they had between-group differences. Where there was an interaction between time and group (*p* < 0.05), post-hoc paired *t*-tests and independent *t*-tests were used to analyze the time effect within each group and between-group difference at each timepoint, respectively. For functional outcomes without a normal distribution, the Friedman test (*post hoc* the Wilcoxon test) and Mann–Whitney U test were used to analyze the time effect within each group and between-group difference at each timepoint, respectively. For variables monitored during the challenging treadmill gait and balance training, the one-way (time) repeated measures ANOVA test was used to analyze the change in tolerated walking speed of gait training and perturbation intensity of balance training across training sessions in the EXP group were used to analyze the change in tolerated walking speed of gait training and perturbation intensity of balance training across training sessions in the EXP group. *p* < 0.05 indicated statistical significance in all analyses.

## 3. Results

**Figure 1:**
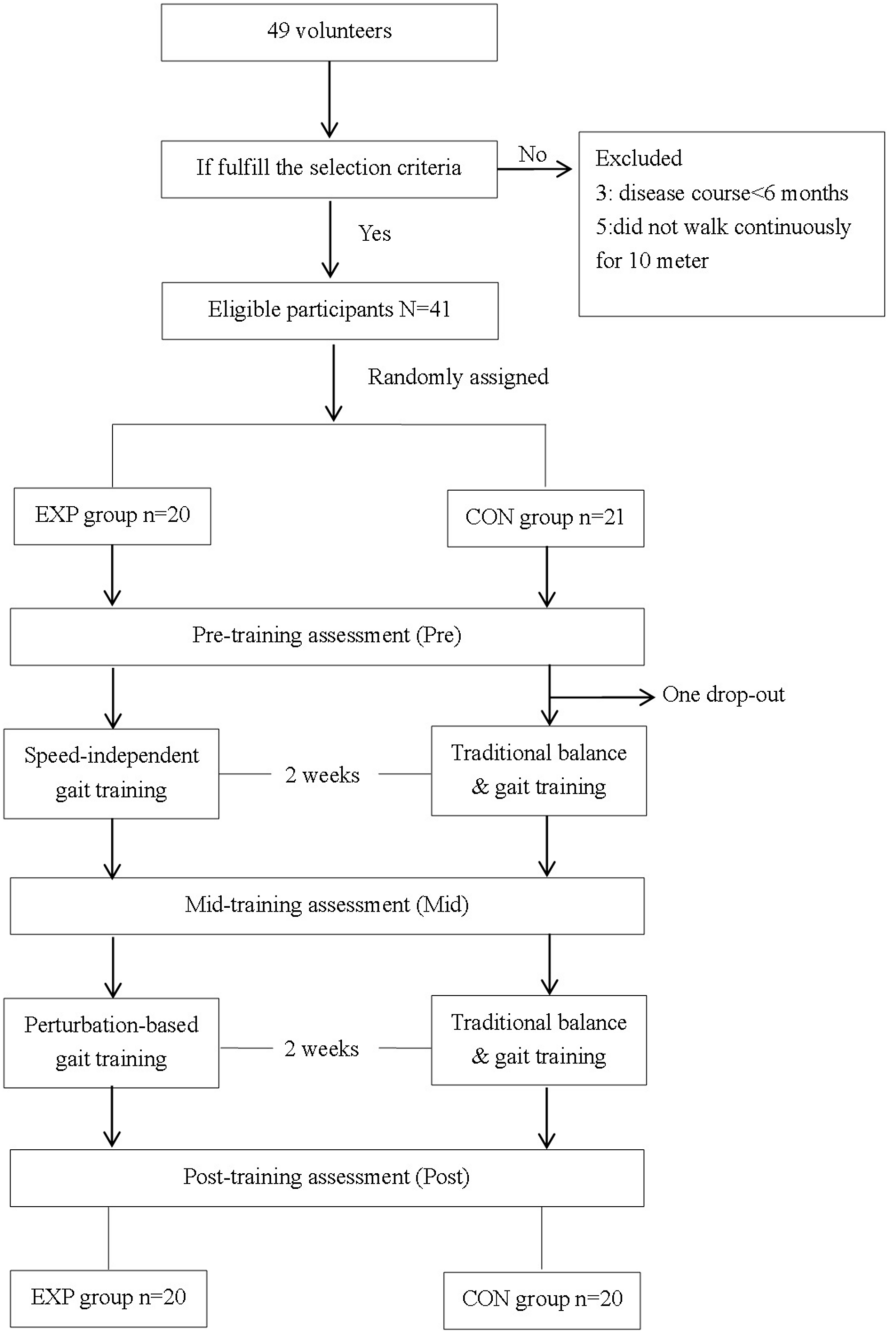
The flow diagram of participants through the study. EXP, experimental group; CON, control group; Pre, pre-training timepoint; Mid, mid-training timepoint; Post, post training timepoint.

A total of 49 patients volunteered for this study, and 16 patients were excluded because they did not meet the inclusion criteria. A total of 33 eligible patients were randomly assigned to either the EXP group (*n* = 17) or the CON group (*n* = 16). All patients completed the 4 week training and three assessments. The flow diagram of participant selection throughout the study is shown in Figure 1. The demographic and disease characteristics were similar between the two groups (*p* > 0.05). There were 29 male and 4 female participants aged 46.0 ± 15.5 years. There were 14 participants that suffered from cerebral infarction and 19 participants that suffered from cerebral hemorrhage at 15.1 ± 16.0 months since stroke upon enrollment. Hemiplegia affected either the right or left side in a ratio of 19:14. The Brunnstrom stage of lower limb ranged from 3 to 6. Nineteen participants used at least one assistive device, including a cane (16 persons) and/or an ankle-foot orthosis (6 persons), in daily life.

The functional outcomes at baseline showed comparable results between the two groups (*p* > 0.05). All participants showed mean gait velocities of 0.34 ± 0.15 m/s and 0.41 ± 0.19 m/s at the normal and fast speeds, respectively. For the reactive balance ability, most participants demonstrated normal response in forward and backward CSTs with rates of 64% and 58%, respectively, whereas a small number of participants (24% and 30%, respectively) lost their balance in the two tests. The participants spent 39.2 ± 13.5 s and 35.0 ± 14.3 s in the TUG test at a normal and fast speed, respectively, and 29.6 ± 11.9 s in the 5-STS test. The average ABC score was 62.7 ± 24.1 out of 100 (Table 2).

**Table 2:** Participants` characteristics and functional outcomes at baseline.

Variables	EXP group (*n* = 17)	CON group (*n* = 16)	*p*
Sex (Male/Female)^a^	14/3	15/1	0.316
Age (years)	48.7 ± 14.8	43.1 ± 16.2	0.304
BMI (kg/m^2^)	25.3 ± 1.7	25.1 ± 3.1	0.796
Type of stroke (infarction/hemorrhage)	6/11	8/8	0.393
Time since stroke (months)	16.2 ± 20.3	14 ± 10.2	0.695
hemiplegia side (Left/Right)^a^	6/11	8/8	0.393
Brunnstrom stages of LE (3/4/5/6)^a^	3/7/5/2	4/9/1/2	0.387
AFO for daily use (Yes/No)^a^	2/15	4/12	0.352
Cane for daily use (Yes/No)^a^	7/10	9/7	0.387
AFO use during tests (Yes/No)^a^	0/17	1/15	0.295
Cane use during tests (Yes/No)^a^	0/17	0/16	–
Gait velocity-normal (m/s)	0.35 ± 0.16	0.32 ± 0.14	0.571
Gait velocity-fast (m/s)	0.43 ± 0.18	0.4 ± 0.2	0.645
CST-Forward (0/1/2/3)^a^	3/0/3/11	5/0/1/10	0.468
CST-Backward (0/1/2/3)^a^	4/0/2/11	6/0/2/8	0.656
TUG_normal (s)	37.5 ± 15.4	41.1 ± 11.3	0.444
TUG_fast (s)	34.2 ± 16	35.9 ± 12.7	0.745
5-STS (s)	28.6 ± 12.6	30.8 ± 11.4	0.608
ABC score	66.5 ± 24.8	58.6 ± 23.3	0.352

*p*, between-group difference examined by the independent *t*-test or Pearson χ^2^ test. BMI, Body mass index; LE, lower extremity; CST, compensatory stepping test; TUG, timed up-and-go test; STS, sit-to-stand test; ABC, the activities-specific balance confidence scale.

a Variables marked with “a” were analyzed by Pearson χ^2^, otherwise by the independent *t*-test.

### 3.1. Safety monitoring and progress of training intensity across challenging gait and balance training sessions in the EXP group

Among the 17 participants in the EXP group, 14 participants completed all gait training sessions, and 13 completed all balance training sessions. No more than two training sessions overall were absent by participants who had not completed all training sessions. Absence was commonly attributed to personal factors or early discharge. The overall completion rate was 95%. Safety analyses were based on available data.

**Figure 2:**
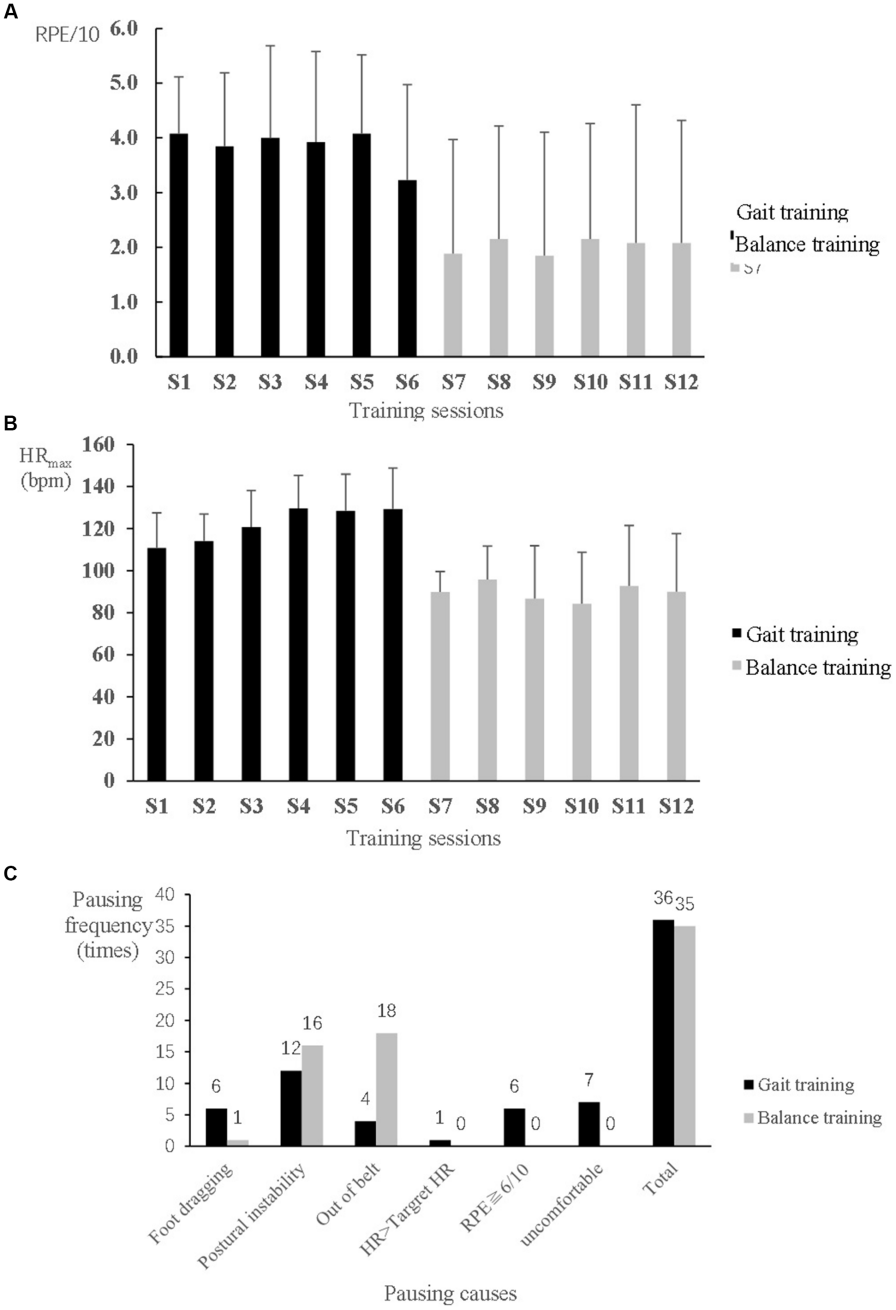
Safety monitoring during speed-dependent gait and perturbation-based balance training in the experimental group. **(A)** Rating of perceived exertion (RPE). **(B)** Maximum heart rate (HR_max_). **(C)** Causes and frequency of pauses during 5 min training phases.

For safety, two participants wore an ankle-foot orthosis on the hemiplegic side during training, one during both gait and balance training and the other one only during balance training. Another participant used ankle taping to improve the stability of the hemiplegic ankle during balance training. The other participants of the EXP group did not use any assistive devices during training. The mean RPE was not over 6/10, and the mean heart rate was not over 130 bpm; both measurements were higher during the gait training than during the balance training (Figures 2A,B). A total of 34 and 20 pauses were given during the gait and balance training phases, respectively, among all EXP participants. During gait training, postural instability, uncomfortable feelings such as spasm or pain around the ankle and foot, foot dragging, and fatigue with over 6/10 of RPE were common causes of pauses. During balance training, stepping out of the belt and postural instability were the main causes of pauses. Resting and reducing the intensity level were commonly useful to avoid or relieve the problems in most participants (Figure 2C).

**Figure 3:**
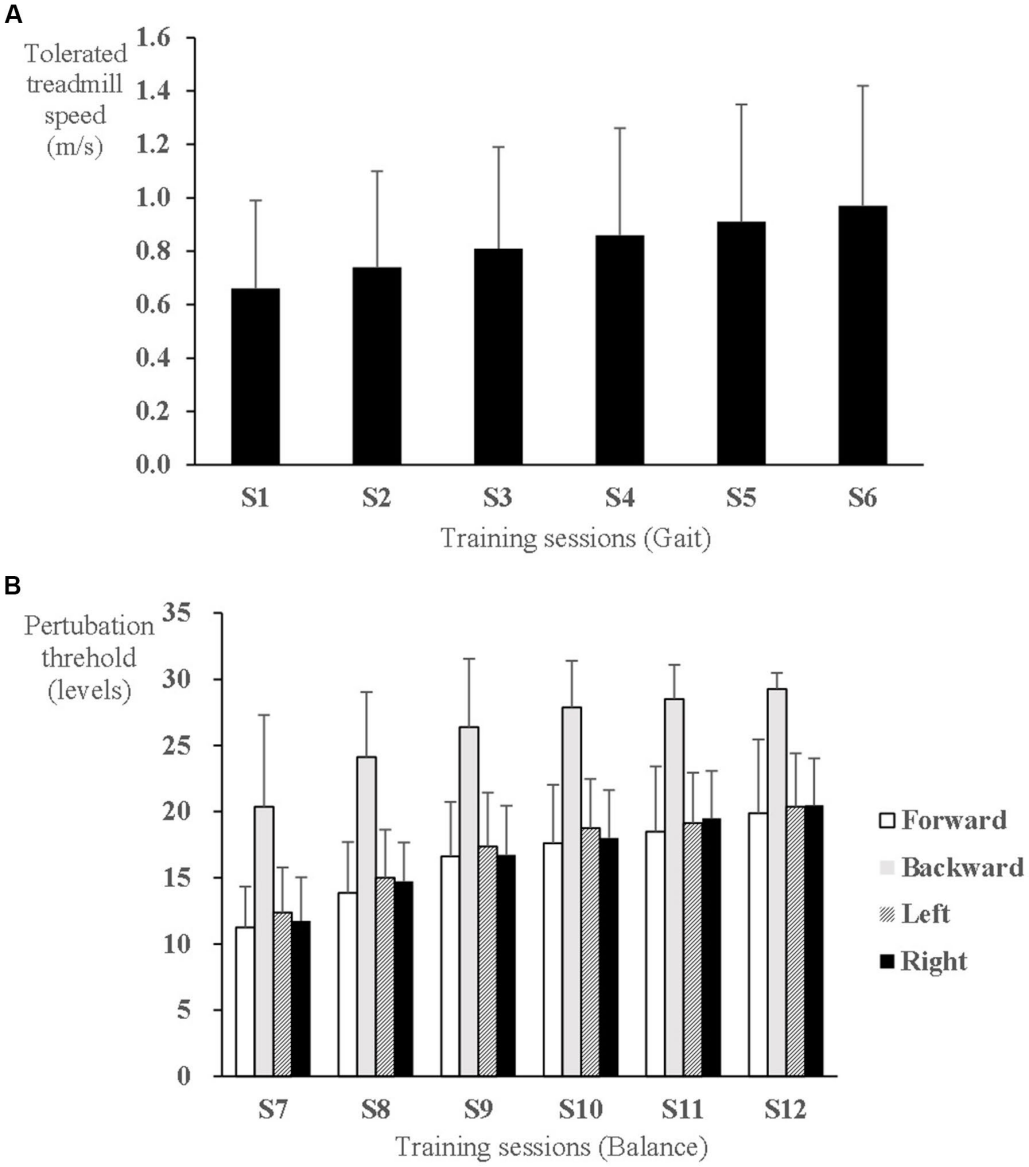
Progress of speed-dependent gait and perturbation-based balance training intensity in the experimental group. **(A)** Tolerated treadmill speed. **(B)** Perturbation threshold to elicit a stepping response.

During gait training, the tolerated treadmill speed significantly increased from 0.56 ± 0.25 m/s at the 1st session to 0.84 ± 0.35 m/s at the 6th session (F_5,65_ = 12.634, *p* < 0.001) (Figure 3A). During balance training, the perturbation threshold was set initially at 11 ± 3, 20 ± 7, 12 ± 3, and 12 ± 4 levels in the direction of forward, backward, left side, and right side, respectively, at the 1st balance training session, which significantly increased to 19 ± 5, 29 ± 1, 20 ± 3, and 20 ± 4 levels, respectively, at the last session (F_5,60_ = 38.796, *p* < 0.001; F_5,60_ = 23.396, *p* < 0.001; F_5,60_ = 32.140, *p* < 0.001; and F_5,60_ = 42.868, *p* < 0.001, respectively) (Figure 3B).

### 3.2. Training effects on gait and balance ability in the EXP group compared with the CON group

For the primary outcomes, the normal gait velocity (F_2,62_ = 3.212, *p* = 0.047) and fast gait velocity (F_2,62_ = 4.252, *p* = 0.019) showed significant time and group interaction effects. Participants in the EXP group showed significant improvement of the normal gait velocity and fast gait velocity after 2 weeks of training (F_1,16_ = 8.539, *p* = 0.010 and F_1,16_ = 13.251, *p* = 0.002, respectively) and 4 weeks of training (F_1,16_ = 9.799, *p* = 0.006 and F_1,16_ = 20.179, *p* < 0.001, respectively) but no improvement in the CON group (*p* > 0.05). There were no significant between-group effects (*p* > 0.05). The reactive balance ability measured by backward CST showed a significant time effect in the CON group (χ^2^_2,16_ = 6.5, *p* = 0.039) but not in the EXP group. There was a significant between-group difference at the Mid timepoint (χ^2^_2,33_ = 6.976, *p* = 0.031) but not at the other two timepoints. The reactive balance ability measured by forward CST did not show any significant time or group effects (*p* > 0.05).

For the secondary outcomes, there were no significant time and group interaction effects or significant group effects (*p* > 0.05). All secondary outcomes demonstrated significant time effects (*p* < 0.05) except for TUG time at a comfortable speed. The EXP group showed a significantly reduced TUG time with fast speed at the Mid (F_1,16_ = 11.221, *p* = 0.004) and Post (F_1,16_ = 12.396, *p* = 0.003) timepoints, whereas the CON group did not show any significant reduction (*p* > 0.05). The EXP group showed a significant reduction in the 5-STS time at the Post timepoint (F_1,16_ = 5.348, *p* = 0.034), whereas the CON group showed similar improvements (F_1,16_ = 5.149, *p* = 0.038). The EXP group significantly increased their ABC score at the Mid timepoint (Z = −2.536, *p* = 0.011) but not at the Post timepoint (*p* > 0.05), whereas the CON group showed no significant increase in ABC score at either timepoint (*p* > 0.05). These details are shown in Table 3.

**Table 3:** Training effects on gait and balance between the EXP and CON groups.

Variables	Groups	Pre	Mid	Post	Time effect (*p*)			Group effect	Time × Group Interaction (*p*)
		Mean ± SD^a^			Overall	Pre vs. Mid	Pre vs. Post	Overall (*p*)	
Gait velocity-normal (m/s)	EXP	0.35 ± 0.16	0.4 ± 0.19	0.42 ± 0.18	0.001*	0.010*	0.006*	0.267	0.047*
	CON	0.32 ± 0.14	0.32 ± 0.14	0.35 ± 0.16		0.633	0.229		
	EXP vs. CON (*p*)	0.571	0.142	0.252					
Gait velocity-fast (m/s)	EXP	0.43 ± 0.18	0.5 ± 0.22	0.51 ± 0.23	<0.001*	0.002*	0.000*	0.319	0.019*
	CON	0.4 ± 0.2	0.4 ± 0.19	0.43 ± 0.21		0.916	0.174		
	EXP vs. CON (*p*)	0.645	0.186	0.274					
CST-Forward (0/1/2/3)^b^	EXP	3/0/3/11	1/0/4/12	1/0/3/13	0.472	0.276	0.194		
	CON	5/0/1/10	3/0/2/11	3/0/4/9	0.368	0.180	0.461		
	EXP vs. CON (*p*)	0.468	0.431	0.398					
CST-Backward (0/1/2/3)^b^	EXP	4/0/2/11	2/0/8/7	2/0/4/11	0.250	0.942	0.391		
	CON	6/0/2/8	4/0/1/11	3/0/1/12	0.039*	0.102	0.059		
	EXP vs. CON (*p*)	0.656	0.031*	0.365					
TUG- normal (s)	EXP	37.46 ± 15.42	37.85 ± 17.68	36.96 ± 16.62	0.216	0.807	0.792	0.413	0.562
	CON	41.13 ± 11.32	43.55 ± 14.91	40.21 ± 13.49		0.211	0.661		
TUG-fast (s)	EXP	34.23 ± 16.02	30.76 ± 15.26	29.28 ± 15.01	<0.001*	0.004*	0.003*	0.496	0.275
	CON	35.89 ± 12.68	35.19 ± 12.81	33.12 ± 12.04		0.633	0.130		
5-STS (s)	EXP	28.58 ± 12.58	25.7 ± 10.76	24.5 ± 10.95	0.001*	0.074	0.034*	0.486	0.896
	CON	30.75 ± 11.42	28.74 ± 10.04	26.8 ± 8.05		0.080	0.038*		
ABC score (0–100)^b^	EXP	66.54 ± 24.82	72.1 ± 21.64	73.46 ± 23.61	0.108	0.011*	0.059		
	CON	58.61 ± 23.32	60.17 ± 23.64	65.74 ± 23.52	0.323	0.937	0.136		
	EXP vs. CON (*p*)	0.410	0.415	0.411					

*p < 0.05. CST, compensatory stepping test; TUG, timed up-and-go test; STS, sit-to-stand test; ABC, the activities-specific balance confidence scale.

a the data are mean ± SD of each variable at each assessment point in each group, except for variables marked with ^b^, which were analyzed using two-way repeated measures ANOVA. For the result of ANOVA, where there was significant time × Group effect, the time effect within each group and the group effect at each assessment point, analyzed by post-hoc tests, are presenting; where there was significant time effect overall, the time effect within each group analyzed by post-hoc tests are presenting in the preset table.

b variables of ordinal data which presents as ratio of each rank at each assessment point in each group, the time and group effects of which were separately analyzed by the Friedman test (post hoc the Wilcoxon test) and Mann–Whitney U test, respectively.

## 4. Discussion

The results demonstrated that the two modes of challenging treadmill-based training, i.e., speed-dependent gait training without body weight support and handrail use, as well as perturbation-induced balance training, are feasible in stroke patients with restricted community ambulation capacity in the chronic phase. In these patients, the challenging treadmill gait and balance training was superior to the traditional training in improving gait velocity while not improving the reactive balance ability, which partially agrees with our hypothesis.

### 4.1. Effects of the 2 week treadmill speed-dependent gait training

The present study newly provides evidence of superior effects on gait velocity improvement of the challenging treadmill gait training without physical assistance compared to traditional gait and balance training in chronic stroke patients with low ambulation ability. The finding is consistent with previous evidence on the effects of speed-dependent gait training with physical assistance in stroke patients newly walking at the subacute stage of recovery (24, 25, 43) and patients with various ambulation levels at the chronic stage (16, 23, 44). The superior effect on improving gait velocity may benefit not only from repetitive practice with a higher number of steps (23, 25) but also from increased levels of biomarkers involved in facilitating neuromuscular plasticity such as lactate and brain-derived neurotrophic factor upregulated through the high-intensity gait training (21, 22). The training intensity was estimated to be at a high level based on the heart rate response during the training of 110–130 bpm reaching ~70–80% of the heart rate reserve.

Moreover, it is worth noting that the training-induced ~20% improvement of gait velocity in our study, exceeding the minimal clinical effect of 0.05 m/s, is higher than the ~10% increase in a previous study with a similar disease course of participants (>12 months after stroke on average) and a similar intervention period (16). It possibly owes to the training without body weight support and handrail use. Without physical assistance, speed-dependent gait training elicited loss of balance in the present study. Fortunately, the incidence rate of this loss of balance was low and avoidable by reducing the treadmill speed. On the whole, the progress of tolerated speed in this study testifies to the feasibility of the training mode in stroke participants with low ambulation ability.

Both TUG time at a fast speed and balance confidence showed improvements after the 2 week speed-dependent gait training but not after traditional training, although without reaching significant group differences. These results indicate that speed-dependent gait training is an efficacious intervention with effect transfer across different activities in chronic patients (34). We noted that TUG improvements only occurred at a fast speed but not a comfortable speed. This can be explained by the similarity principle of learning transfer (45), TUG at a fast speed has relatively more similarity in speed features with the speed-dependent gait training, compared to that at a comfortable speed, thereby gaining more improvement from the training. With longer training duration, the TUG time at a comfortable speed may also improve, as shown in previous studies (16, 24).

### 4.2. Effects of 4 week challenging treadmill gait and balance training

This study is the first to combine treadmill-based speed-dependent gait training with perturbation-induced balance training in chronic stroke patients, and this approach has comparable balance and gait training components of the traditional approach. We found superior effects of the mixed treadmill-based gait and balance training on improving gait velocity and TUG performance at a fast speed than the traditional gait and balance training. However, we did not detect additional benefit from the later 2 week perturbation-induced balance training on basis of the earlier 2 week treadmill-based training benefit. In the present study, due to the high similarity in treadmill gait training components between the two training modes of the EXP group, a generalization (46) effect could be produced from the first 2 week training and determine the similar gain during the second 2 week. Besides, carry-over effect (47) from the first 2 week training would merge into the gain during the second 2 week training. The study design makes it impossible to isolate the effects of perturbation-induced balance training on ambulation function in the stroke patients of this study, which need studies with a true experimental design to clarify.

Regarding reactive balance ability, we detected progress in the perturbation threshold of the stepping responses across the treadmill gait perturbation training sessions that indicates improvement in skills to cope with gait perturbations. However, we did not find an improvement in the skills in response to stance perturbations measured by CST tests after a 4 week challenging treadmill-based training. Based on the specificity principle (12), these results could be attributed to a lack of relevant stance perturbation training in the training protocol. It hints at the possible absence of a transfer of acquired skills from gait perturbations to stance perturbations. Hence, perturbation-based training while standing or during walking may both need to be included in the training protocol targeting reactive balance ability. Furthermore, the manner of delivery of multidirectional perturbations might be a factor influencing acquisition of skills to cope with perturbations. This study adopted a mixed delivery of perturbations with reference to the protocols of previous studies (28, 32). However, a recent study by Dusane and Bhatt found that mixed perturbations in opposite directions interfere with motor adaptation, particularly slip-response adaptation (48). They suggested that block training of each perturbation might better enhance reactive postural responses than the mixed perturbation training in stroke patients (48). Hence, interference from the mixed delivery of perturbations might weaken the acquisition of skills from gait perturbation, making the acquisition not strong enough for skill transfer. Further studies in stroke patients are needed to compare the effects of perturbation-induced training in blocks with those in a random sequence.

Regarding the 5-STS, both the EXP and CON groups showed new improvement after 4 week gait and balance training. The 5-STS reflects not only dynamic balance ability but also strength of the anti-gravity muscles (49). Previous studies reported increased quadriceps strength in stroke patients after treadmill gait training (50) or treadmill perturbation-induced balance training (28, 32). Therefore, the improvement in 5-STS performance may be attributed to the increased strength of the quadriceps muscles by challenging treadmill gait and balance training in the EXP group and may result from increased balance ability by transferring task-specific training effects in the CON group.

Regarding balance confidence, the significant improvement gained in the EXP group from treadmill gait training was not maintained during the treadmill perturbation-induced balance training. This finding is inconsistent with those of recent studies showing significant improvements in balance confidence after treadmill perturbation-induced balance training (28, 32). Noting that the mean ABC scores were comparable between the 2 week and 4 week timepoints while the training effects differed, we further checked the changes in ABC scores from the baseline and intermediate timepoints to the end timepoint and found several outliers in the changes in ABC scores. Most outliers were positive changes apart from one negative shift (Supplementary Figure S1), which increased the variation in changes and hid the positive effects of treadmill perturbation-induced balance training on balance confidence. Moreover, the ambulation ability of participants in this study was much lower than that in the abovementioned previous studies with treadmill perturbation-induced balance training (mean comfortable gait velocity at baseline: 0.32 m/s vs. 0.8–0.9 m/s). For those participants with lower ambulation ability, treadmill perturbation-induced balance training was particularly challenging. When responding well in challenging training situations, the participants were usually very excited and became very confident in coping with daily activities, otherwise they became very depressed or scared weakening their confidence, thereby leading to the observed large variance. In general, the effect of treadmill perturbation-induced balance training on improving balance confidence could be as promising as the treadmill speed-dependent gait training. Safe training protocols and positive feedbacks are important to overcome negative psychological impacts.

In summary, this study established the feasibility of the two strategies of challenging treadmill speed-dependent gait training and perturbation-induced balance training in chronic stroke patients with low ambulation ability by monitoring safety and evaluating the effects of the trainings on ambulation. However, this study has some limitations. First, no measure directly reflecting reactive balance ability during ambulation was adopted to investigate the training effects. Second, the study design did not allow to clarify the effectiveness of treadmill perturbation-induced balance training alone as carryover and generalization effects perturbed the results. Third, the final sample size of this study met the requirement for detecting significant differences in gait velocity changes between the EXP and CON groups. However, the age of participants in the two groups had noticeably difference and variable range (EXP: 48.7±14.8 and CON: 43.1±16.24), although without statistical between-group differences. It is a small sample size leading to inadequate statistical power to detect statistically differences (51, 52). Besides, the sample size limited us from exploring the feasibility and effectiveness of the training within different age ranges of participants. A larger sample size is required for further studies clarifying the effectiveness of each training mode with different age ranges. Fourth, it is not known whether one group had a disproportionate number of individuals with cognitive impairment, and this could have been a confounding factor in interpreting the results. One inclusion criterion we set for participants was their ability to follow commands: this was a minimum prerequisite for the completion of study tasks by participants. However, this participant selection criterion did not require the overall intactness of cognitive functions and instead was meant to ensure that more volunteers would be eligible to enroll in our study. Fifth, we did not track fall events after treatment. Despite an increased gait velocity in the EXP group, these patients still had balance impairments with a mean TUG of ~37 s and no improved skill to cope with stance perturbation. Higher mobility but poor balancing ability may lead to a greater exposure to fall-prone environments or activities, thereby contributing conversely to a higher risk of falls for patients with chronic stroke (53). Further studies are needed on how changes in gait velocity and balance ability affect falls after treatment.

## 5. Conclusion

The two training strategies of challenging treadmill speed-dependent gait and perturbation-induced balance training, without weight support and use of handrails, are feasible and effective in improving the ambulation function in patients with chronic stroke who have low ambulation ability.

## Data availability statement

The original contributions presented in the study are included in the article/Supplementary material, further inquiries can be directed to the corresponding authors.

## Ethics statement

The studies involving human participants were reviewed and approved by Medical Ethics Committee of the Guangdong Work Injury Rehabilitation Center (AF/SC-07/2016.01, Chinese Clinical Trials.gov registration number ChiCTR-IOR-16009536). The patients/participants provided their written informed consent to participate in this study.

## Author contributions

JH: conceptualization, investigation, formal analysis, funding acquisition, and writing – original draft. LJ: resources, critical review and approval of manuscript. YW: resources, supervision, and writing – review and editing. XS: conceptualization, methodology, resources, supervision, funding acquisition, and writing – review and editing. All authors contributed to the article and approved the submitted version.

## Funding

This work was supported by the Medical Scientific Research Foundation of Guangdong Province of China (grant number, 201617224333897) and Shanghai Sports Science Research Foundation (grant number, 19 T004).

## Conflict of interest

The authors declare that the research was conducted in the absence of any commercial or financial relationships that could be construed as a potential conflict of interest.

## Publisher’s note

All claims expressed in this article are solely those of the authors and do not necessarily represent those of their affiliated organizations, or those of the publisher, the editors and the reviewers. Any product that may be evaluated in this article, or claim that may be made by its manufacturer, is not guaranteed or endorsed by the publisher.
