# Supplementary material for: Feasibility of challenging treadmill speed-dependent gait and perturbation-induced balance training in chronic stroke patients with low ambulation ability: a randomized controlled trial
Source: Front Neurol. 2023 Jul 17;14:1167261. doi: 10.3389/fneur.2023.1167261 (PMC10389716; doi:10.3389/fneur.2023.1167261)
Supplement: Supplementary file 1 [file Image_1.pdf]

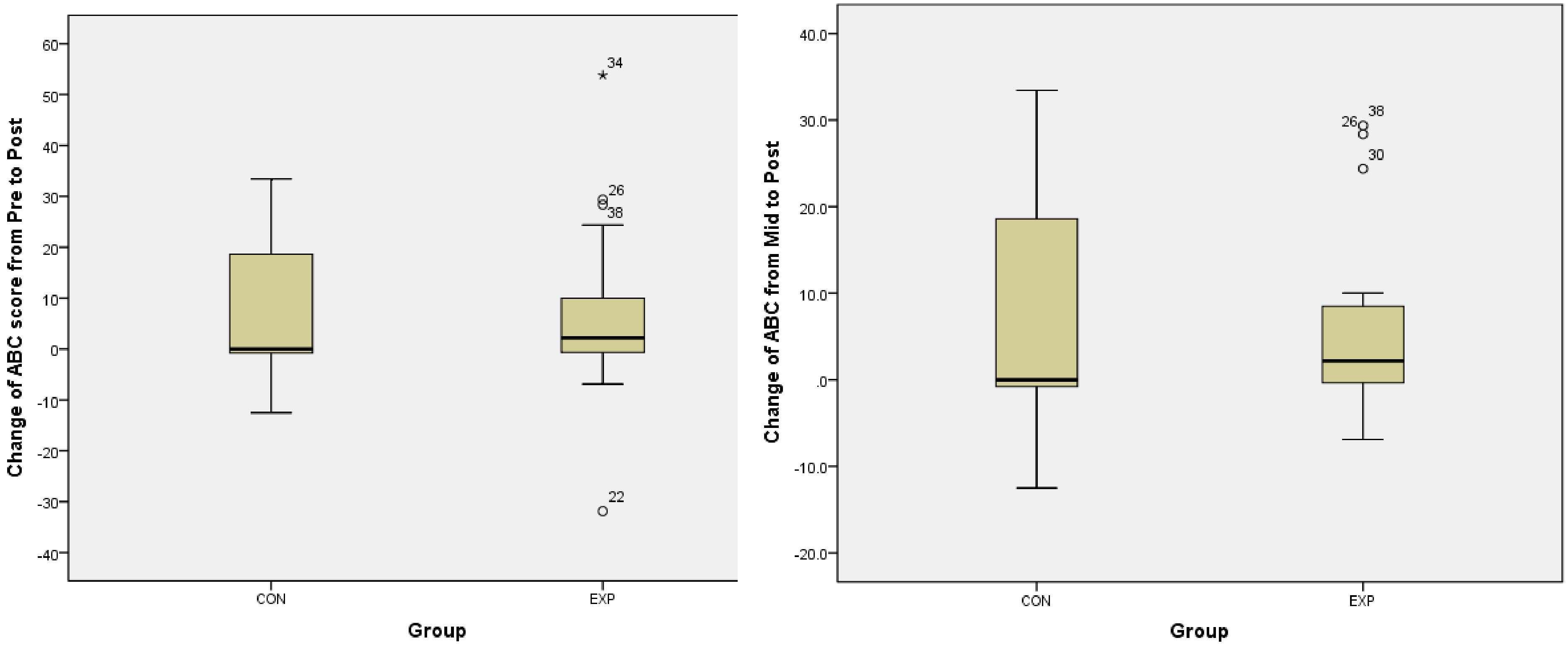

Supplementary Figure 1. Change of ABC score after 4-week of training in the experimental group (EXP) and control group (CON)

ABC: the activities-specific balance confidence scale
